# Beyond Survival: Factors Driving Textbook Outcome After Simultaneous Pancreas–Kidney Transplantation—A Retrospective Analysis

**DOI:** 10.3390/jcm15041465

**Published:** 2026-02-13

**Authors:** Anke Mittelstädt, Frederik Weber, Maximilian Brunner, Christian Krautz, Florian Struller, Hendrik Apel, Bernd Wullich, Katharina Heller, Mirian Opgenoorth, Mario Schiffer, Robert Grützmann, Georg F. Weber

**Affiliations:** 1 Department of General and Visceral Surgery, Friedrich-Alexander-University (FAU), Krankenhausstraße 12, 91054 Erlangen, Germany; frederik@webermc.de (F.W.); maximilian.brunner@uk-erlangen.de (M.B.); christian.krautz@uk-erlangen.de (C.K.); robert.gruetzmann@uk-erlangen.de (R.G.); 2 General and Visceral Surgery, Krankenhaus Wasserburg, Gabersee 1, 83512 Wasserburg am Inn, Germany; florian.struller@ro-med.de; 3 Department of Urology and Pediatric Urology, Friedrich-Alexander-University (FAU), Krankenhausstraße 12, 91054 Erlangen, Germany; hendrik.apel@uk-erlangen.de (H.A.); bernd.wullich@uk-erlangen.de (B.W.); 4 Department of Nephrology and Hypertension, Friedrich-Alexander-University (FAU), 91054 Erlangen, Germany; katharina.heller@uk-erlangen.de (K.H.); mirian.opgenoorth@uk-erlangen.de (M.O.); mario.schiffer@uk-erlangen.de (M.S.)

**Keywords:** simultaneous pancreas–kidney transplantation, textbook outcome, donor selection, cold ischemia time, perioperative morbidity, graft function, survival analysis

## Abstract

**Background:** Simultaneous pancreas–kidney transplantation (SPK) is the standard treatment for selected patients with type 1 diabetes mellitus and end-stage renal disease. Textbook Outcome (TO), a composite of perioperative and long-term quality indicators, provides a benchmark for optimal results. This study analyzed factors associated with failure to achieve TO after SPK. **Methods:** We retrospectively analyzed 119 SPK recipients (1980–2022). TO was defined according to IQTIG criteria: (i) patient survival ≥ 3 years, (ii) insulin independence at discharge, (iii) kidney function at discharge (GFR ≥ 20 mL/min), (iv) insulin-free survival ≥ 3 years, and (v) sustained kidney function ≥ 3 years. Predictors of TO failure were identified by logistic regression. Long-term survival was assessed by Kaplan–Meier analysis. **Results:** Ninety-two patients were eligible for TO assessment; 52% achieved TO. Compared with TO patients, non-TO patients had older donors (median 30 vs. 25.5 years, *p* = 0.017), older recipients (44 vs. 39 years, *p* = 0.012), longer kidney cold ischemia time (CIT; 13.0 vs. 9.7 h, *p* = 0.005), and more pancreatic complications (*p* = 0.009). In multivariate analysis, donor age (OR 1.050, *p* = 0.030) and kidney CIT (OR 1.180, *p* = 0.029) independently predicted TO failure. Cut-offs were donor age ≤ 37 years and kidney CIT ≤ 11.5 h. Patients achieving TO had significantly better long-term survival (15 years, *p* = 0.0077). **Conclusions:** Younger donor age and shorter kidney CIT independently predict TO achievement, which is associated with superior long-term survival. Optimized donor selection and perioperative management may improve SPK outcomes.

## 1. Introduction

Simultaneous pancreas-kidney transplantation (SPK) is the treatment of choice for selected patients with type 1 diabetes mellitus (T1DM) and end-stage renal disease (ESRD), offering the unique potential to restore normoglycemia and renal function in a single procedure. The primary goal is to achieve long-term glycemic control and to prevent or even reverse diabetes-related complications, particularly micro- and macrovascular pathologies [1,2]. Since the first successful pancreas transplantation in 1966 [3], advances in surgical techniques, immunosuppression, and perioperative care have led to significantly improved outcomes, including patient survival rates exceeding 90% at one year and sustained insulin independence in most recipients [4,5].

In 2022, 44 pancreas transplantations were performed in Germany across 17 centers, with a slight decline in transplant numbers and waitlist registrations since 2013 [6]. This decrease is partly due to improved diabetes and nephrology care delaying dialysis and transplantation need. Despite fewer procedures, outcomes have improved, with one-year survival after transplantation significantly higher than on the waitlist [5,7,8]. SPK transplantation provides better and more physiological glycemic control than insulin therapy or islet transplantation and prevents diabetic nephropathy in the transplanted kidney [9]. It also improves peripheral circulation, neuropathy, and cardiovascular risk factors, significantly enhancing patients’ quality of life [10,11]. SPK remains the gold standard for type 1 diabetic patients with nephropathy, offering the only long-term normoglycemia [1].

Despite improved outcomes after SPK, pancreas transplantation remains the solid organ transplant with the highest complication rate. Early postoperative complications are predominantly surgical, while immunological complications, such as rejection, tend to occur later [6,12]. Pancreatic graft thrombosis, particularly venous, is a leading cause of early graft loss and requires prompt diagnosis and intervention [13,14]. Transplant pancreatitis varies in severity and may necessitate graft explantation in fulminant cases [15,16]. Postoperative bleeding can originate from anastomotic sites or the graft itself and may require endoscopic or surgical treatment [14,16].

To optimize outcomes in SPK, risk factors must be minimized, and procedures should be limited to experienced high-volume centers with close interdisciplinary follow-up care [17]. Two scoring systems are currently used to assess donor suitability: the Preprocurement Pancreas Suitability Score (P-PASS), based solely on donor parameters [18], and the Pancreas Donor Risk Index (PDRI), which includes additional transplant-related variables and predicts one-year graft survival [18,19]. Donor age, cold ischemia time, and donor characteristics such as obesity, metabolic disturbances, and organ appearance significantly affect graft outcomes [20,21,22,23]. Additionally, HLA mismatches, particularly at HLA-A, -B, and -DR loci, are associated with increased rejection risk; a higher number of mismatches correlates with poorer transplant outcomes [24].

A Textbook Outcome (TO) represents an ideal postoperative course by combining multiple outcome and quality indicators into a single, standardized measure [25]. These typically include short-term mortality, absence of complications, length of hospital stay, and organ function. First introduced by Kolfschoten et al. in 2012 for colon resections [26], TO has since been applied across various fields of complex visceral surgery, including liver transplantation [27], bariatric surgery [28], retroperitoneal sarcomas [29], and pancreatic surgery [30], as a comprehensive measure of treatment quality.

The aim of this study was to identify potential factors associated with failure to achieve a Textbook Outcome as defined by IQTIG criteria (Patient survival ≥ 3 years; no need for insulin at discharge; adequate kidney function at discharge [GFR ≥ 20 mL/min]; insulin-free period ≥ 3 years; sustained kidney function [GFR ≥ 20 mL/min] for ≥3 years) [31] and to compare these with established risk factors and donor- and recipient-specific characteristics. The goal is to gain new insights into donor and recipient selection in order to reduce the rate of perioperative complications and improve the likelihood of achieving a Textbook Outcome in the future. Given the lack of comprehensive data on textbook outcome–based quality metrics in SPK, particularly in the context of national quality indicators, this study was designed as an exploratory, hypothesis-generating analysis to identify potentially modifiable factors associated with failure to achieve a textbook outcome.

## 2. Materials and Methods

A retrospective, single-center observational study was conducted at University Hospital Erlangen. For each patient, the observation period extended from the time of transplantation to the date of data collection or the last available clinical follow-up.

All patients who underwent SPK at the center between 1980 and 2022 were considered for inclusion. In total, 119 patients met the initial inclusion criteria. Of these, 27 patients were excluded due to missing data necessary for the assessment of TO. Patients who received isolated pancreas transplants or islet cell transplants were not included in the analysis.

The study was approved by the Ethics Committee of Friedrich-Alexander University Erlangen-Nürnberg (FAU) following thorough review (approval number 23-423-Br).

**Surgical technique**

All procedures were performed under general anesthesia with the patient in the supine position. Midline laparotomy was performed. The right iliac vessels and the inferior vena cava were exposed and prepared for vascular anastomoses. Following systemic heparinization, a tangential cavotomy was performed, and the pancreas graft—prepared on the back table—was anastomosed via porto-cavostomy to the inferior vena cava and via an arterio-arterial anastomosis (Y-graft of the superior mesenteric and splenic artery) to the right iliac artery. Duodenojejunostomy was performed in a side-to-side fashion.

Kidney transplantation followed via retroperitoneal approach to the left iliac vessels. Venous and arterial anastomoses were performed to the external iliac vein and the common iliac artery, respectively. Ureteroneocystostomy with antireflux tunnel technique and stent insertion was completed. Final inspection included hemostasis and Doppler sonographic verification of adequate graft perfusion.

**Data collection**

Data on recipient and donor demographics, comorbidities, preoperative parameters, transplantation details, and postoperative course were collected and analyzed. The primary outcome was the identification of risk factors associated with failure to achieve a textbook outcome. In addition, long-term survival was assessed.

**Determination of Textbook Outcome Status**

The following criteria were used to define the presence of a textbook outcome:

- Patient survival ≥ 3 years
- No need for insulin therapy at the time of discharge
- Adequate renal function at the time of discharge (GFR ≥ 20 mL/min)
- Insulin-free survival for ≥3 years
- Adequate renal function (GFR ≥ 20 mL/min) maintained for ≥3 years

If any of these quality criteria were not met, the case was classified as “no textbook outcome.” The selection of these criteria was based on the current quality indicators of the Institute for Quality Assurance and Transparency in Healthcare (IQTIG) for pancreas and combined pancreas-kidney transplantation [31].

Patients were excluded from TO assessment if essential data required for the evaluation of one or more TO components were missing. These cases could therefore not be reliably classified as TO or non-TO.

**Statistical analysis**

Data analysis was performed using SPSS software, version 28.0 (IBM Corp., Armonk, NY, USA), and GraphPad Prism, version 10 (GraphPad Software, San Diego, CA, USA). Continuous and ordinal variables were compared using the Student’s *t*-test or the Mann–Whitney U test, as appropriate. Categorical variables were analyzed using the chi-square test. A *p*-value ≤ 0.05 was considered statistically significant.

All relevant clinical and perioperative parameters were evaluated as potential risk factors for failure to achieve a textbook outcome using univariate analysis. To ensure a clear conceptual separation between predictors and outcome-defining variables, only perioperative pancreatic complications occurring within 30 days after transplantation were considered as potential predictors of TO failure. Outcome components and direct consequences of TO failure, such as insulin dependence, graft explantation, or graft loss, were not included as predictors in order to avoid circular reasoning. Variables with a *p*-value ≤ 0.1 in univariate analysis were included in the multivariate analysis.

For variables identified as statistically significant, the minimum *p*-value approach with Bonferroni correction was applied to determine optimal cut-off values associated with the respective outcome parameters.

Survival analyses were performed using the Kaplan–Meier method. Group differences in survival were assessed using the log-rank test.

All parameters were analyzed at single, predefined time points. Longitudinal survival was assessed using Kaplan–Meier analysis; no repeated measurements within subjects were performed.

Missing data were handled using a complete-case approach for each analysis; therefore, sample sizes varied across analyses depending on data availability. Survival analyses included all patients with available follow-up data.

## 3. Results

### 3.1. Recipients and Donors in the Non-TO Group Were Significantly Older

A significant age difference between the TO and non-TO groups was observed, with recipients in the non-TO group having a higher median age (44 years) compared to the TO group (39 years; *p* = 0.012) (Table 1). No other significant differences in baseline demographic characteristics were noted. CMV serostatus, transplantation waiting time, dialysis-related parameters, comorbidities, and laboratory values were comparable between the two groups.

Donors in the non-TO group were also significantly older than those in the TO group, with a median age of 30 years versus 25.5 years (*p* = 0.017) (Table 1). No significant differences were found regarding donor BMI, gender, CMV serostatus, cause of death, laboratory results, or resuscitation status.

While kidney function at discharge (including serum creatinine and the presence of a functioning kidney) did not differ significantly between groups, achievement of a textbook outcome depends on the fulfillment of all predefined composite criteria. Accordingly, patients could meet individual discharge parameters yet fail to achieve TO due to other components, such as lack of insulin independence or insufficient long-term graft function.

### 3.2. Cold Ischemia Time of the Kidney Was Shorter in the TO Group

Analysis of transplant-related parameters revealed that the cold ischemia time (CIT) of the kidney was significantly longer in the non-TO group compared to the TO group (median 13.0 vs. 9.7 h; *p* = 0.005) (Table 1). All other ischemia times, HLA mismatches, donor-recipient gender mismatch, CMV mismatch (positive donor to negative recipient), and duration of surgery did not differ significantly between the groups.

### 3.3. Non-TO Patients Experienced More Pancreatic Complications and a Higher Clavien-Dindo Score

Postoperative outcomes showed a significantly higher incidence of perioperative pancreatic complications in the non-TO group compared to the TO group (52% vs. 26%; *p* = 0.009). Early pancreatic graft dysfunction occurred more frequently in the non-TO group (4 vs. 0 patients; *p* = 0.030), as did early kidney graft dysfunction (6 vs. 0 patients; *p* = 0.007). Clavien-Dindo complication scores were significantly higher in the non-TO group (*p* = 0.003). Long-term pancreatic complications were also more common in the non-TO group (79% vs. 47%; *p* = 0.002), whereas no differences were observed in long-term kidney-related complications. Immunosuppressive regimens were comparable between both groups (Table 1).

According to the group classification, a significantly higher proportion of patients in the non-TO group were not insulin-independent at discharge (21% vs. 0%; *p* < 0.001), underwent early pancreas (*p* < 0.001) and kidney explantation (*p* = 0.033), and experienced pancreas graft loss (21 vs. 8 patients; *p* = 0.001) (Table 1).

There were no significant differences between the groups in terms of serum creatinine levels at discharge, need for postoperative dialysis, kidney graft loss, length of hospital stay, or in-hospital mortality.

### 3.4. Donor Age and Prolonged Cold Ischemia Time of the Kidney Are Independent Risk Factors for Failure to Achieve a TO

In the univariate analysis of pre- and intraoperative factors associated with failure to achieve a TO, higher recipient age (*p* = 0.015), higher donor age (*p* = 0.019), and longer cold ischemia time (CIT) of the kidney (*p* = 0.008) were identified as significant risk factors. In the multivariate logistic regression analysis, both higher donor age (OR = 1.050; *p* = 0.030) and prolonged CIT of the kidney (OR = 1.180; *p* = 0.029) remained independent predictors for not achieving a TO (Table 2). Although recipient age was significantly associated with TO failure in univariate analysis, this association did not remain significant after multivariate adjustment, indicating that recipient age was not an independent predictor.

Using an exploratory minimum *p*-value approach, optimal cut-off values were identified: a donor age > 37 years (*p* = 0.000454; Bonferroni-corrected *p* = 0.0181) (Table 3a) and a CIT of the kidney > 11.5 h (*p* = 0.000103; Bonferroni-corrected *p* = 0.0067) were significantly associated with a failure to achieve a TO (Table 3b).

### 3.5. Perioperative Pancreatic Complications Reduce the Likelihood of Achieving TO

When analyzed individually, perioperative pancreatic complications were significantly more frequent among patients who failed to achieve a textbook outcome, indicating a strong association with non-TO status (OR 3.208, Table 4). As only one postoperative variable reached statistical significance, a multivariate analysis of postoperative factors was not performed.

### 3.6. Reasons for Failure to Achieve a Textbook Outcome

**Figure 1:**
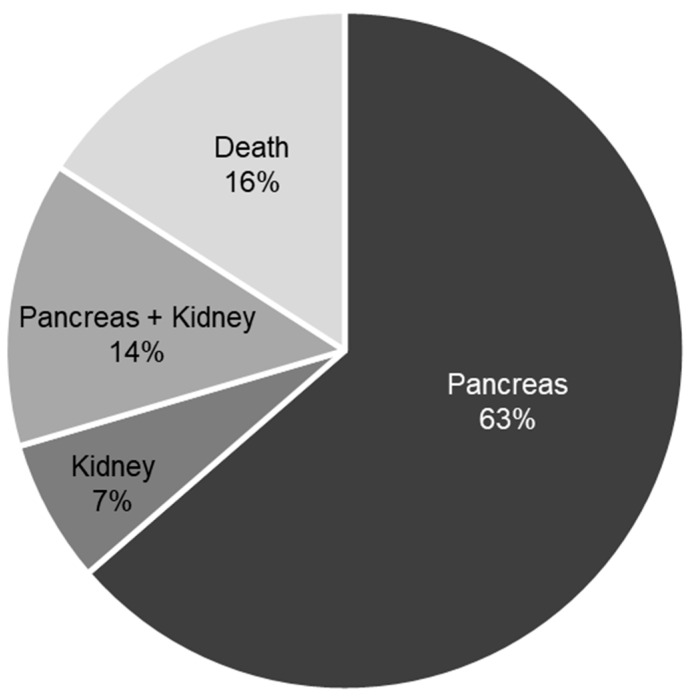
Reasons for TO failure.

Figure 1 illustrates the reasons for failure to achieve a textbook outcome. The most frequent cause was pancreas graft failure or loss of insulin independence, accounting for 63% of non-TO cases. Sixteen percent of patients in the non-TO group died within the first three years after transplantation. Combined dysfunction of both pancreas and kidney grafts was observed in 14% of cases. Isolated kidney graft failure occurred least frequently and was identified in only 7% of patients who did not achieve a TO.

### 3.7. Patients Achieving a TO Have Significantly Improved Long-Term Survival

**Figure 2:**
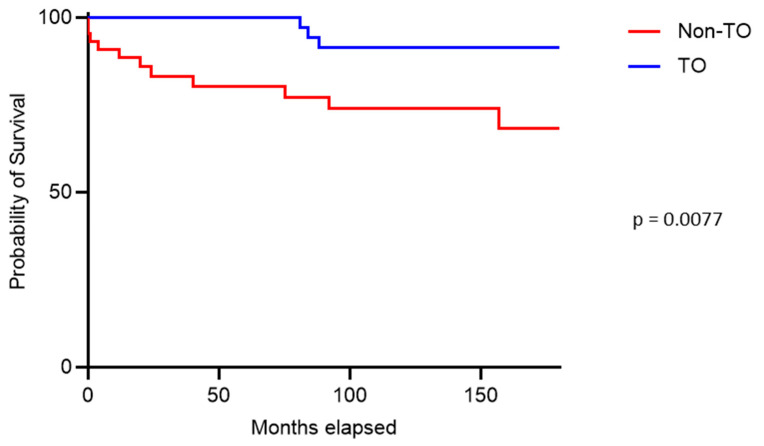
Patient survival.

In the survival analysis, patients who achieved a TO demonstrated significantly longer overall survival after a 15-year follow-up period (*p* = 0.0077) (Figure 2).

## 4. Discussion

In this study, we analyzed clinical and perioperative differences between patients who achieved a textbook outcome (TO) and those who did not, and we identified independent risk factors associated with failure to achieve a TO.

Recipient age and body mass index (BMI) are key factors in the evaluation process for pancreas–kidney transplantation and play a central role in determining transplant eligibility and waitlisting. In our cohort, the mean recipient age was 41 years. Although recipients with a TO were significantly younger than those without, multivariate analysis did not confirm recipient age as an independent risk factor associated with failure to achieve a TO. The impact of recipient age on post-transplant outcomes has been reported inconsistently in the literature. Messner et al. [32] found in a dual-center study that older recipients exhibited lower overall survival compared to younger recipients. This was attributed to increased frailty due to longer-standing diabetic metabolic conditions and the associated late complications of diabetes. However, pancreatic and renal graft function was comparable between older and younger recipients [32]. Since our study investigated the achievement of a textbook outcome (TO) rather than overall survival, direct comparability is limited. Nevertheless, recipient age was a risk factor for failure to achieve a TO in univariate analysis. Other authors have reported equivalent survival rates of patients, pancreas, and kidney grafts across age groups [33,34]. Montagud-Marrahi et al. concluded that diabetes duration, rather than chronological age, had a relevant impact on survival and was particularly associated with an increased incidence of major cardiovascular events [35]. No differences in recipient BMI were observed between the groups, which can be attributed to the exclusion of patients with elevated BMI from transplantation eligibility.

In the present study, we demonstrated that higher donor age was associated with a significantly lower likelihood of achieving a TO. Multivariate analysis revealed that each additional year of donor age increased the odds of failing to achieve a TO by 5% (OR = 1.05). Using the minimum *p*-value approach, we identified a cut-off value of 37 years for donor age. Among patients receiving organs from donors aged ≤37 years, 42 of 66 (64%) achieved a TO, whereas only 6 of 26 patients (23%) achieved a TO when donor age exceeded 37 years. The donor age remains one of the most controversial factors in SPK. A number of studies have identified increased donor age as a key risk factor for perioperative complications and early graft loss [21,36,37]. In an analysis by Humar et al., donor age emerged as the most significant predictor of pancreatic graft thrombosis [37]. In a comprehensive literature review published in 2020, Muñoz-Bellvís and López-Sánchez reported that donor age > 45 years was associated with a modestly increased risk, while age > 50 years was associated with a markedly elevated risk of complications and reduced graft survival [20]. The persistent shortage of standard or “ideal” donors in both Germany and abroad further complicates organ allocation. In 2022, 47 out of 54 pancreas transplant offers were declined at our center due to insufficient organ quality. This raises the ongoing debate about expanding donor criteria, particularly with respect to increasing the acceptable donor age. Bonham et al. argued that donor age alone is not necessarily linked to increased risk, but that accurate assessment of organ quality by an experienced transplant surgeon plays a decisive role [38]. Similarly, a study by Salvalaggio et al. [36] showed that although grafts from older donors (>45 years) had higher failure rates, transplantation still conferred a survival benefit compared to remaining on the waiting list. They concluded that using organs from donors older than 45 years may improve overall survival of waitlisted patients, even though outcomes are inferior to those from younger donors [36]. Direct comparison with our findings is limited, as our study focused on achieving a textbook outcome rather than isolated organ-specific complications. However, when considering overall outcomes—including postoperative pancreatic and renal function and patient survival—as reflected by the composite TO metric, our data suggest that donor age may have a more substantial impact than previously assumed, challenging recent trends in donor acceptance.

Our data demonstrated a significant difference in kidney cold ischemia time (CIT) between the TO and non-TO groups. The median CIT was 3.3 h shorter in the TO group. In multivariate analysis, kidney CIT was identified as an independent risk factor for failure to achieve a TO. Using the minimum *p*-value approach, we defined a cutoff value of 11.5 h. Among patients with a kidney CIT exceeding this threshold, only 10 out of 35 achieved a TO. There are limited data available on kidney CIT in the context of SPK. Most studies focus on kidney CIT in isolated kidney transplantation. In general, prolonged CIT has been associated with an increased incidence of delayed graft function and primary non-function, as reported by multiple authors [39,40,41]. However, the kidney is considerably more tolerant to ischemia compared to the pancreas. In kidney transplantation, CITs of up to 40 h are considered acceptable [39,40,41]. In SPK, since both organs are typically transplanted into the same recipient, kidney CIT usually exceeds pancreas CIT by only a few hours. In our study, the median kidney CIT was 10.8 h, approximately one hour longer than the pancreas CIT. The potential impact of kidney CIT on outcomes in SPK should be further evaluated in prospective studies. The identified cut-off values for donor age and kidney cold ischemia time were derived using a post-hoc, data-driven approach and should therefore be interpreted with caution. Although a Bonferroni correction was applied to reduce the risk of type-I error, these thresholds are exploratory in nature and require validation in independent, preferably prospective cohorts.

In contrast, we did not observe a significant association between pancreas cold ischemia time (CIT) and the achievement of a TO. The median pancreas CIT in our cohort was 10 h, which lies within the range generally considered acceptable [2]. Pancreas CIT is often regarded as a relevant factor in pancreas transplantation outcomes due to its role in ischemia-reperfusion injury. Drognitz et al. demonstrated that such injury induces apoptosis of acinar cells, thereby impairing microcirculation [42]. However, they also emphasized that the choice of preservation solution may have a greater impact on graft viability than the duration of cold ischemia itself. Current literature and guidelines issued by the German Medical Association recommend limiting pancreas CIT to a maximum of 12 h [2,20]. The lack of association observed in our study may be attributed to the relatively short ischemia times in our cohort. Rudolph et al. similarly noted that studies failing to demonstrate a significant influence of pancreas CIT on outcomes typically reported mean CITs between 8 and 13 h [43]. This suggests that within this time frame, the impact of pancreas CIT on TO in this cohort may be limited.

Perioperative pancreatic complications emerged as an important determinant of failure to achieve a textbook outcome. Patients who developed pancreas-related perioperative complications were substantially less likely to achieve an ideal postoperative outcome, even when this factor was examined in isolation. In our cohort, such complications were almost twice as frequent in the non-TO group and were associated with higher rates of early graft dysfunction—both pancreatic and renal—as well as higher overall morbidity, reflected by significantly elevated Clavien–Dindo scores. The sustained difference in long-term pancreatic complications, despite comparable immunosuppressive regimens, suggests that early pancreatic events may have lasting consequences for graft function. The association between perioperative pancreatic complications and failure to achieve TO should be interpreted descriptively rather than causally. Given the central role of early pancreatic events in determining subsequent graft function and insulin independence, such complications represent an expected correlate of non-TO status rather than an independent mechanistic driver. Accordingly, this finding does not provide novel mechanistic insight but underscores the internal consistency of the composite outcome definition. Similar observations have been reported by other authors, who identified early technical complications—particularly thrombosis—as leading causes of graft loss and reduced survival in pancreas transplantation [13,44]. Likewise, Fridell et al. emphasized that preventing early surgical complications is critical for long-term success, as initial pancreatic graft injury may predispose to chronic dysfunction and recurrent complications [45]. These findings underscore the importance of meticulous intraoperative technique, early detection, and prompt management of pancreatic complications to maximize the likelihood of achieving optimal outcomes.

Whereas the textbook outcome includes kidney-related criteria, failure to achieve TO in our cohort was not predominantly driven by renal dysfunction. In line with clinical experience in SPK transplantation, pancreas-related failure—particularly loss of insulin independence or pancreas graft failure—represented the most frequent limiting factor [12,13,14,15]. In contrast, isolated kidney graft failure accounted for only a small proportion of non-TO cases. These findings support the interpretation of TO as a global quality indicator that primarily reflects the complexity of sustained pancreas graft function rather than a kidney-driven outcome.

Although HLA mismatches are considered relevant in pancreas transplantation [24], no significant association with textbook outcome was observed in our cohort. Given the exploratory design and limited sample size, the study was not powered to detect small to moderate effects of HLA mismatching, and the absence of an association should therefore be interpreted with caution.

Patient and graft survival are key outcome parameters in SPK [31]. In a multivariate analysis by Gruessner et al., the function of the transplanted organs was identified as a crucial determinant of patient survival [46]. Particularly, early pancreas graft loss has been shown to negatively impact patient survival [47,48,49,50]. The German Institute for Quality Assurance and Transparency in Healthcare (IQTIG) defines benchmark survival rates of ≥90% at 1 year, ≥80% at 2 years, and ≥75% at 3 years post-transplantation [31]. In our center, we observed patient survival rates of 95.8%, 93.0%, and 91.1% at 1, 2, and 3 years, respectively. These survival rates exceed the minimum benchmark thresholds defined by IQTIG and reflect the outcomes achieved at a high-volume tertiary referral center. However, given the single-center design and the relatively limited sample size, our data do not support revising established national benchmark criteria. Rather, they suggest that optimized patient selection, perioperative management, and center experience may allow outcomes to surpass current benchmark requirements. We aligned the definition of Textbook Outcome (TO) in our study with these national quality benchmarks, incorporating both patient and graft survival up to three years post-transplantation. Notably, medium- and long-term patient survival was significantly higher in the TO group compared to the non-TO group. Strikingly, in the TO group, the first deaths occurred only after seven years, whereas survival probability declined steadily in the non-TO group. Although achievement of a textbook outcome requires survival of at least three years and thus introduces an inherent guarantee-time bias, patients achieving TO demonstrated a consistently higher long-term survival probability. When restricting the analysis to patients alive at three years, survival differences were no longer statistically significant, likely reflecting limited statistical power after landmarking. Nevertheless, the observed survival pattern supports an association between achieving a textbook outcome and favorable long-term prognosis rather than a causal survival benefit. Our findings suggest that TO is not only associated with favorable short-term outcomes, but also with improved medium- and long-term survival, which is consistent with observations from previous studies [25,27,30].

The present study was designed as an exploratory, hypothesis-generating analysis. While the identified associations may help to highlight potentially modifiable factors related to the achievement of a textbook outcome, the findings should not be interpreted as practice-changing and require confirmation in prospective, multicenter studies. This study represents a single-center retrospective analysis, which may limit the generalizability of the results to other centers or regions. Furthermore, due to the long time span covered by the data, the quality and completeness of documentation—particularly in earlier cases—was limited. As a result, not all relevant variables were available for every patient, and the sample size (N) varied across analyses due to case-wise exclusions. Additionally, the long inclusion period inevitably encompasses substantial changes in surgical technique, organ preservation, and immunosuppressive regimens. Due to the limited sample size, a formal adjustment for transplant era was not performed, and potential era effects should therefore be considered when interpreting the results. In particular, earlier transplant eras were more likely to be associated with longer cold ischemia times and higher perioperative complication rates, which may have contributed to the observed associations between donor age, kidney cold ischemia time, and failure to achieve a textbook outcome. Finally, the retrospective nature of the study precludes causal conclusions, limiting interpretation to associative relationships.

## 5. Conclusions

Our findings demonstrate that in simultaneous pancreas–kidney transplantation, younger donor age and shorter kidney cold ischemia time are independent determinants for achieving a TO, which itself is associated with improved long-term survival. These results underscore the necessity of stringent donor selection criteria and optimized perioperative coordination to maximize the probability of an optimal postoperative course. Given the ongoing shortage of suitable organs, careful balancing of donor age thresholds against the urgency of transplantation remains crucial. Future prospective, multicenter studies are warranted to validate these cut-off values, integrate them into donor allocation algorithms, and explore whether targeted interventions to reduce kidney ischemia time can further enhance outcomes in this high-risk patient population.

## Figures and Tables

**Table 1:** Clinical data in association with Textbook Outcome (TO).

	All Patients	TO	non-TO	*p*
**Recipients**				
Number, N(%)	92 (100)	48 (52)	44 (48)	
**Age (years), median (IQR)**	**41 (11)**	**39 (8)**	**44 (16)**	**0.012**
BMI (kg/m^2^), median (IQR) (n = 91) *	23.5 (5.3)	22.8 (5.5)	24.1 (5)	0.162
Gender (male), n (%)	59 (64)	29 (60)	30 (68)	0.438
Tx waiting time (months) (IQR) (n = 90) *	13 (11)	14 (9)	12 (13)	0.400
CMV status positive, n (%) (n = 86) *	43 (50)	23 (50)	20 (50)	1.000
Dialysis				
Duration of preop. dialysis (months) (IQR) (n = 91) *	18 (22)	18 (21)	18 (28)	0.880
Haemodialysis, n (%) (n = 90) *	66 (73)	37 (79)	29 (67)	0.227
Peritoneal dialysis, n (%) (n = 90) *	17 (19)	8 (17)	9 (21)	0.636
Haemo- and Peritoneal dialysis, n (%) (n = 90) *	5 (6)	2 (4)	3 (7)	0.573
No dialysis before Tx (preemptive), n (%) (n = 91) *	13 (14)	5 (10)	8 (19)	0.265
Comorbidities, n (%)				
Peripheral arterial disease (PAD) (n = 83) *	22 (27)	12 (29)	10 (24)	0.666
Coronary artery disease (CAD) (n = 84) *	31 (37)	14 (33)	17 (42)	0.389
Bypass/Stent (n = 84) *	16 (19)	8 (19)	8 (20)	0.916
Pelvic axis calcification (n = 82) *	41 (50)	20 (47)	21 (54)	0.507
Retinopathy (n = 88) *	76 (86)	40 (87)	36 (86)	0.865
Neuropathy (n = 88) *	60 (68)	30 (62)	30 (71)	0.532
Laboratory results before Tx, median (IQR)				
HbA1c (%) (n = 63) *	7.5 (2.3)	7.8 (2.8)	7.4 (1.7)	0.248
Total cholesterol (mg/dL) (n = 72) *	187 (55)	188 (57)	187 (74)	0.320
HDL-cholesterol (mg/dL) (n = 66) *	53 (23)	53 (22)	52.5 (30)	0.484
LDL-cholesterol (mg/dL) (n = 66) *	116.5 (45)	115 (52)	122 (39)	0.214
Creatinine (mg/dL) (n = 78) *	6.5 (4)	6.4 (4.2)	6.6 (3.7)	0.775
**Donors**				
**Age (years), median (IQR)**	**27 (20)**	**25.5 (15)**	**30 (25)**	**0.017**
BMI (kg/m^2^), median (IQR)	23.5 (4.6)	23.2 (5.3)	23.9 (4.3)	0.956
Gender (male), n (%)	56 (61)	31 (65)	25 (57)	0.446
CMV status positive, n (%) (n = 91) *	38 (42)	20 (43)	18 (41)	0.874
Cause of death, n (%)				
Intracranial hemorrhage (n = 91) *	45 (50)	22 (47)	23 (52)	0.602
Traumatic brain injury (TBI) (n = 91) *	32 (35)	16 (34)	16 (36)	0.817
Cardiovascular, stroke, anoxic brain injury (n = 91) *	9 (10)	5 (11)	4 (9)	0.805
Primary brain tumor (n = 91) *	1 (1)	1 (2)	0 (0)	0.331
Meningitis/Encephalitis (n = 91) *	2 (2)	1 (2)	1 (2)	0.962
Others (n = 91) *	2 (2)	2 (4)	0 (0)	0.166
Laboratory results, median (IQR)				
Sodium (mmol/L) (n = 90) *	149 (10)	150 (13)	148 (9)	0.362
Lipase (U/mL) (n = 67) *	34 (41)	36 (46)	27 (35)	0.478
Amylase (U/L) (n = 71) *	68 (58)	74 (77)	60 (44)	0.160
Glucose (mg/dL) (n = 88) *	144 (59)	159 (90)	138 (74)	0.097
Resusciation, n (%) (n = 91) *	7 (8)	3 (6)	4 (9)	0.585
**Transplantation**				
Ischemia times, median (IQR)				
Cold ischemia time (CIT) pancreas (h) (n = 88) *	10 (4)	8.8 (4.3)	10 (2.7)	0.396
Cold ischemia time (CIT) kidney (h) (n = 84) *	**10.8 (4.6)**	**9.7 (2.4)**	**13 (3.7)**	**0.005**
Warm ischemia time (WIT) pancreas (min) (n = 62) *	38 (16)	36 (14)	42.5 (17)	0.164
Warm ischemia time (WIT) kidney (min) (n = 84) *	40 (21)	40 (22)	39 (17)	0.957
HLA Mismatches (MMbroad), median (IQR) (n = 85) *	4 (1)	4 (2)	5 (1)	0.319
CMV positiv → negativ, n (%) (n = 86) *	18 (21)	10 (22)	8 (20)	0.843
Transmission by gender, n (%)				
female → male	28 (30)	13 (27)	15 (34)	0.466
female → female	8 (9)	4 (8)	4 (9)	0.898
male → female	25 (27)	15 (31)	10 (23)	0.359
male → male	31 (34)	16 (33)	15 (34)	0.939
Duration of surgery (min), median (IQR) (n = 67) *	377 (158)	369 (161)	380 (156)	0.869
**Postoperative**				
Perioperative complications pancreas (≤30 days), n (%) (n = 89) *	**34 (38)**	**12 (26)**	**22 (52)**	**0.009**
Thrombosis	13 (15)	4 (9)	9 (21)	0.085
Rejection	6 (7)	1 (2)	5 (12)	0.066
Pancreatitis	17 (19)	8 (17)	9 (21)	0.597
Bleeding	6 (7)	4 (9)	2 (5)	0.481
**Graft dysfunction**	**4 (5)**	**0 (0)**	**4 (10)**	**0.030**
Infection/abscess	5 (6)	1 (2)	4 (10)	0.130
Anastomotic leakage	2 (2)	0 (0)	2 (5)	0.130
Perioperative complications kidney (≤30 days), n (%) (n = 90) *	33 (37)	15 (31)	18 (43)	0.254
Thrombosis	10 (11)	6 (13)	4 (10)	0.654
Rejection	20 (22)	10 (21)	10 (24)	0.735
Bleeding	5 (6)	2 (4)	3 (7)	0.539
**Kidney failure**	**6 (7)**	**0 (0)**	**6 (14)**	**0.007**
Infection	2 (2)	1 (2)	1 (2)	0.924
Clavien-Dindo, n (%) (n = 89) *				**0.003**
I	0 (0)	0 (0)	0 (0)	
II	19 (21)	13 (28)	6 (14)	
III	19 (21)	11 (23)	8 (19)	
IV	12 (14)	0 (0)	12 (29)	
V	2 (2)	0 (0)	2 (5)	
Long-term complications pancreas, n (%) (n = 89) *	**55 (62)**	**22 (47)**	**33 (79)**	**0.002**
Thrombosis	13 (15)	4 (9)	9 (21)	0.085
Rejection	18 (20)	8 (17)	10 (24)	0.426
Pancreatitis	20 (23)	9 (19)	11 (26)	0.427
Bleeding	6 (7)	4 (9)	2 (5)	0.481
Graft dysfunction	12 (14)	4 (9)	8 (19)	0.146
Infection/abscess	6 (7)	1 (2)	5 (12)	0.066
Anastomotic leakage	3 (3)	0 (0)	3 (7)	0.062
Long-term complications kidney, n (%) (n = 90) *	63 (70)	33 (69)	30 (71)	0.782
Thrombosis	12 (14)	6 (13)	6 (15)	0.769
Rejection	38 (43)	21 (44)	17 (41)	0.828
Bleeding	5 (6)	2 (4)	3 (7)	0.536
Kidney failure	16 (18)	7 (15)	9 (22)	0.367
Infection	5 (6)	2 (4)	3 (7)	0.520
Immunosuppression, n (%) (n = 75) *				
Induction therapy	75 (100)	37 (100)	38 (100)	
IL2-RA	31 (41)	14 (38)	17 (45)	0.544
ATG	44 (59)	23 (62)	21 (55)	0.544
Insulin-free at discharge, n (%) (n = 87) *	**56 (64)**	**48 (100)**	**8 (21)**	**<0.001**
Creatinine at discharge (mg/dL), median (IQR)	1.25 (0.5)	1.2 (0.5)	1.34 (0.6)	0.228
Primary functioning kidney, n (%) (n = 85) *	63 (69)	38 (81)	25 (66)	0.115
Postoperative dialysis, n (%)	16 (17)	9 (19)	7 (16)	0.720
Functioning kidney at discharge, n (%) (n = 85) *	80 (94)	46 (98)	34 (90)	0.102
**Early pancreas explantation (** **≤** **30 days), n (%)**	**11 (12)**	**0 (0)**	**11 (25)**	**<0.001**
**Early kidney explantation (** **≤** **30 days), n (%)**	**4 (4)**	**0 (0)**	**4 (9)**	**0.033**
**Pancreas graft loss, n (%)**	**29 (32)**	**8 (17)**	**21 (48)**	**0.001**
Kidney graft loss, n (%)	21 (23)	11 (23)	10 (23)	0.983
Duration of hospital stay (days), median (IQR) (n = 87) *	26 (20)	29 (16)	23 (25)	0.905
In-hospital death, n (%) (n = 89) *	2 (2)	0 (0)	2 (5)	0.122

(n = 92), * = missing data (n < 92), TO = Textbook Outcome, CAD = coronary artery disease, PAD = peripheral arterial disease (n = 92).

**Table 2:** Association of pre- and intraoperative factors with Textbook Outcome (TO).

	non-TO vs. TO			
	Univariate	Multivariate		
	*p*	OR	95% CI	*p*
**Recipients**				
**Age (years)**	**0.015**	1.060	0.998–1.127	0.59
BMI (kg/m^2^)	0.162			
Gender (male)	0.439			
Duration of preop. Dialysis (months)	0.632			
No dialysis before Tx (preemptive)	0.271			
Tx waiting time (months)	0.500			
Pelvic axis calcification	0.507			
**Donors**				
**Age (years)**	**0.019**	**1.050**	**1.005–1.097**	**0.030**
BMI (kg/m^2^)	0.955			
Gender (male)	0.446			
Sodium (mmol/L)	0.359			
Lipase (U/mL)	0.235			
Amylase (U/mL)	0.152			
Resusciation	0.588			
**Transplantation**				
Cold ischemia time (CIT) pancreas (h)	0.392			
**Cold ischemia time (CIT) kidney (h)**	**0.008**	**1.180**	**1.017–1.368**	**0.029**
Warm ischemia time (WIT) pancreas (min)	0.168			
Warm ischemia time (WIT) kidney (min)	0.949			
HLA mismatches MMbroad	0.329			
Transmission female → male	0.466			
Transmission female → female	0.898			
Transmission male → female	0.360			
Transmission male → male	0.939			
Duration of surgery (min)	0.866			

**Table 3:** (**a**) Minimum *p*-value approach (TO and Donor Age); (**b**) Miminum *p*-value approach (TO and CIT kidney).

**(a)**			
	**Textbook Outcome (TO)**		**Total**
	**No**	**Yes**	
Age Donor ≤ 37	24	42	66
Age Donor > 37	20	6	26
Total	44	48	92
Chi-square-test: ***p* = 0.000454**, after Bonferroni correction ***p* = 0.0181**, n = 92			
**(b)**			
	**Textbook Outcome (TO)**		**Total**
	**No**	**Yes**	
CIT kidney ≤ 11.5 h	14	35	49
CIT kidney > 11.5 h	25	10	35
Total	39	45	84
Chi-square-test: ***p* = 0.000103**, after Bonferroni correction ***p* = 0.0067**, n = 84			

**Table 4:** Association of postoperative factors with Textbook Outcome (TO).

	non-TO vs. TO		
	Univariate		
	*p*	OR	95% CI
**Postoperative**			
Insulin-free at discharge	0.997		
Primary functioning kidney	0.119		
Postoperative dialysis	0.720		
Functioning kidney at discharge	0.139		
**Perioperative complications pancreas**	**0.010**	**3.208**	**1.314–7.832**
Perioperative complications kidney	0.256		
Duration of hospital stay (days)	0.223		
In-hospital death	0.999		

## Data Availability

The data presented in this study are available within the article. No additional data were generated or analyzed.

## Abbreviations

The following abbreviations are used in this manuscript:

BMI	Body Mass Index
CI	Confidence Interval
CIT	Cold Ischemia Time
CMV	Cytomegalovirus
ESRD	End-Stage Renal Disease
FAU	Friedrich-Alexander University Erlangen–Nürnberg
GFR	Glomerular Filtration Rate
HLA	Human Leukocyte Antigen
IBM	International Business Machines Corporation
IQTIG	Institute for Quality Assurance and Transparency in Healthcare
OR	Odds Ratio
PDRI	Pancreas Donor Risk Index
P-PASS	Preprocurement Pancreas Suitability Score
SPK	Simultaneous Pancreas–Kidney Transplantation
SPSS	Statistical Package for the Social Sciences
T1DM	Type 1 Diabetes Mellitus
TO	Textbook Outcome

## References

[1] Dholakia S., Royston E., Quiroga I., Sinha S., Reddy S., Gilbert J., Friend P.J. (2017). The rise and potential fall of pancreas transplantation. Br. Med. Bull..

[2] Bundesärztekammer (2022). Neubekanntmachung der Richtlinie Gem. § 16 Abs. 1 S. 1 Nrn. 2 u. 5 TPG für die Wartelistenführung und die Organvermittlung zur Pankreastransplantation und Kombinierten Pankreas-Nierentransplantation.

[3] Kelly W.D., Lillehei R.C., Merkel F.K., Idezuki Y., Goetz F.C. (1967). Allotransplantation of the pancreas and duodenum along with the kidney in diabetic nephropathy. Surgery.

[4] Gruessner A.C., Gruessner R.W.G. (2018). Pancreas Transplantation for Patients with Type 1 and Type 2 Diabetes Mellitus in the United States: A Registry Report. Gastroenterol. Clin. N. Am..

[5] Rössler F., de Rougemont O. (2022). Update zur Pankreastransplantation. Die Diabetol..

[6] Deutsche Stiftung Organtransplantation. https://www.dso.de/organspende/statistiken-berichte/organtransplantation.

[7] Reddy K.S., Stablein D., Taranto S., Stratta R.J., Johnston T.D., Waid T.H., McKeown J.W., Lucas B.A., Ranjan D. (2003). Long-term survival following simultaneous kidney-pancreas transplantation versus kidney transplantation alone in patients with type 1 diabetes mellitus and renal failure. Am. J. Kidney Dis..

[8] Alhamad T., Malone A.F., Brennan D.C., Stratta R.J., Chang S.H., Wellen J.R., Horwedel T.A., Lentine K.L. (2017). Transplant Center Volume and the Risk of Pancreas Allograft Failure. Transplantation.

[9] Gerber P.A., Pavlicek V., Demartines N., Zuellig R., Pfammatter T., Wüthrich R., Weber M., Spinas G.A., Lehmann R. (2008). Simultaneous islet-kidney vs pancreas-kidney transplantation in type 1 diabetes mellitus: A 5 year single centre follow-up. Diabetologia.

[10] Aly M.G., Morath C., Mehrabi A., Zeier M. (2020). Was gibt es Neues zur kombinierten Nieren-Pankreas-Transplantation?. Der Nephrol..

[11] Smets Y.F., Westendorp R.G., van der Pijl J.W., de Charro F.T., Ringers J., de Fijter J.W., Lemkes H.H. (1999). Effect of simultaneous pancreas-kidney transplantation on mortality of patients with type-1 diabetes mellitus and end-stage renal failure. Lancet.

[12] Wullstein C., Woeste G., Taheri A.S., Dette K., Bechstein W.O. (2003). Morbidity following simultaneous pancreas/kidney transplantation. Chirurg.

[13] Ai Li E., Farrokhi K., Zhang M.Y., Offerni J., Luke P.P., Sener A. (2023). Heparin Thromboprophylaxis in Simultaneous Pancreas-Kidney Transplantation: A Systematic Review and Meta-Analysis of Observational Studies. Transpl. Int..

[14] Vincent M., Morla O., Branchereau J., Karam G., Dupas B., Frampas E. (2014). Multi detector computed tomography (MDCT) for the diagnosis of early complications after pancreas transplantation. Abdom. Imaging.

[15] Manrique A., Jiménez C., López R.M., Cambra F., Morales J.M., Andrés A., Gutiérrez E., Ortuño T., Calvo J., Sesma A.G. (2009). Relaparotomy after pancreas transplantation: Causes and outcomes. Transplant. Proc..

[16] Büttner-Herold M., Amann K., Pfister F., Tannapfel A., Maslova M., Wunsch A., Pillokeit N., Viebahn R., Schenker P. (2021). Pancreas transplantation-clinic, technique, and histological assessment. Pathologe.

[17] Schenker P., Viebahn R. (2013). Perspective of simultaneous pancreas-kidney transplantation. Dtsch. Med. Wochenschr..

[18] Ayami M.S., Grzella S., Kykalos S., Viebahn R., Schenker P. (2018). Pancreas Donor Risk Index but Not Pre-Procurement Pancreas Allocation Suitability Score Predicts Pancreas Graft Survival: A Cohort Study from a Large German Pancreas Transplantation Center. Ann. Transplant..

[19] Axelrod D.A., Sung R.S., Meyer K.H., Wolfe R.A., Kaufman D.B. (2010). Systematic evaluation of pancreas allograft quality, outcomes and geographic variation in utilization. Am. J. Transplant..

[20] Muñoz-Bellvís L., López-Sánchez J. (2020). Donor risk factors in pancreas transplantation. World J. Transplant..

[21] Douzdjian V., Gugliuzza K.G., Fish J.C. (1995). Multivariate analysis of donor risk factors for pancreas allograft failure after simultaneous pancreas-kidney transplantation. Surgery.

[22] Kandaswamy R., Stock P.G., Gustafson S.K., Skeans M.A., Urban R., Fox A., Odorico J.S., Israni A.K., Snyder J.J., Kasiske B.L. (2019). OPTN/SRTR 2017 Annual Data Report: Pancreas. Am. J. Transplant..

[23] Humar A., Ramcharan T., Kandaswamy R., Gruessner R.W., Gruessner A.C., Sutherland D.E. (2004). Technical failures after pancreas transplants: Why grafts fail and the risk factors--a multivariate analysis. Transplantation.

[24] Rudolph E.N., Dunn T.B., Mauer D., Noreen H., Sutherland D.E., Kandaswamy R., Finger E.B. (2016). HLA-A, -B, -C, -DR, and -DQ Matching in Pancreas Transplantation: Effect on Graft Rejection and Survival. Am. J. Transplant..

[25] van der Kaaij R.T., de Rooij M.V., van Coevorden F., Voncken F.E.M., Snaebjornsson P., Boot H., van Sandick J.W. (2018). Using textbook outcome as a measure of quality of care in oesophagogastric cancer surgery. Br. J. Surg..

[26] Kolfschoten N.E., Kievit J., Gooiker G.A., van Leersum N.J., Snijders H.S., Eddes E.H., Tollenaar R.A., Wouters M.W., Marang-van de Mheen P.J. (2013). Focusing on desired outcomes of care after colon cancer resections; hospital variations in ‘textbook outcome’. Eur. J. Surg. Oncol..

[27] Moris D., Shaw B.I., Gloria J., Kesseli S.J., Samoylova M.L., Schmitz R., Manook M., McElroy L.M., Patel Y., Berg C.L. (2020). Textbook Outcomes in Liver Transplantation. World J. Surg..

[28] Poelemeijer Y.Q.M., Marang-van de Mheen P.J., Wouters M., Nienhuijs S.W., Liem R.S.L. (2019). Textbook Outcome: An Ordered Composite Measure for Quality of Bariatric Surgery. Obes. Surg..

[29] Wiseman J.T., Ethun C.G., Cloyd J.M., Shelby R., Suarez-Kelly L., Tran T., Poultsides G., Mogal H., Clarke C., Tseng J. (2020). Analysis of textbook outcomes among patients undergoing resection of retroperitoneal sarcoma: A multi-institutional analysis of the US Sarcoma Collaborative. J. Surg. Oncol..

[30] van Roessel S., Mackay T.M., van Dieren S., van der Schelling G.P., Nieuwenhuijs V.B., Bosscha K., van der Harst E., van Dam R.M., Liem M.S.L., Festen S. (2020). Textbook Outcome: Nationwide Analysis of a Novel Quality Measure in Pancreatic Surgery. Ann. Surg..

[31] Institute for Quality Assurance and Transparency in Health Care (IQTIG) Qualitätsindikatoren für Pankreas- und Pankreas-Nierentransplantationen. https://iqtig.org/qs-verfahren/pntx/.

[32] Messner F., Leemkuil M., Yu Y., Massie A.B., Krendl F.J., Benjamens S., Bösmüller C., Weissenbacher A., Schneeberger S., Pol R.A. (2021). Recipient age and outcome after pancreas transplantation: A retrospective dual-center analysis. Transpl. Int..

[33] Scalea J.R., Redfield R.R., Arpali E., Leverson G., Sollinger H.W., Kaufman D.B., Odorico J.S. (2016). Pancreas transplantation in older patients is safe, but patient selection is paramount. Transpl. Int..

[34] Laurence J.M., Marquez M.A., Seal J.B., Sapisochin G., Bazerbachi F., Selzner M., Norgate A., Greig P.D., McGilvray I.D., Schiff J. (2015). The effect of recipient age on outcome after pancreas transplantation. Transplantation.

[35] Montagud-Marrahi E., Molina-Andújar A., Pané A., Ramírez-Bajo M.J., Amor A., Esmatjes E., Ferrer J., Musquera M., Diekmann F., Ventura-Aguiar P. (2020). Outcomes of pancreas transplantation in older diabetic patients. BMJ Open Diabetes Res. Care.

[36] Salvalaggio P.R., Schnitzler M.A., Abbott K.C., Brennan D.C., Irish W., Takemoto S.K., Axelrod D., Santos L.S., Kocak B., Willoughby L. (2007). Patient and graft survival implications of simultaneous pancreas kidney transplantation from old donors. Am. J. Transplant..

[37] Humar A., Kandaswamy R., Granger D., Gruessner R.W., Gruessner A.C., Sutherland D.E. (2000). Decreased surgical risks of pancreas transplantation in the modern era. Ann. Surg..

[38] Bonham C.A., Kapur S., Dodson S.F., Dvorchik I., Corry R.J. (1999). Potential use of marginal donors for pancreas transplantation. Transplant. Proc..

[39] Lum E.L., Homkrailas P., Abdalla B., Danovitch G.M., Bunnapradist S. (2023). Cold Ischemia Time, Kidney Donor Profile Index, and Kidney Transplant Outcomes: A Cohort Study. Kidney Med..

[40] Debout A., Foucher Y., Trébern-Launay K., Legendre C., Kreis H., Mourad G., Garrigue V., Morelon E., Buron F., Rostaing L. (2015). Each additional hour of cold ischemia time significantly increases the risk of graft failure and mortality following renal transplantation. Kidney Int..

[41] Sampaio M.S., Chopra B., Tang A., Sureshkumar K.K. (2018). Impact of cold ischemia time on the outcomes of kidneys with Kidney Donor Profile Index ≥85%: Mate kidney analysis—A retrospective study. Transpl. Int..

[42] Drognitz O., Obermaier R., Liu X., Neeff H., von Dobschuetz E., Hopt U.T., Benz S. (2004). Effects of organ preservation, ischemia time and caspase inhibition on apoptosis and microcirculation in rat pancreas transplantation. Am. J. Transplant..

[43] Rudolph E.N., Dunn T.B., Sutherland D.E.R., Kandaswamy R., Finger E.B. (2017). Optimizing outcomes in pancreas transplantation: Impact of organ preservation time. Clin. Transplant..

[44] Kjøsen G., Rydenfelt K., Horneland R., Aandahl E.M., Line P.D., Dorenberg E., Berstad A.E., Brabrand K., Hagen G., Pischke S.E. (2021). Early detection of complications in pancreas transplants by microdialysis catheters, an observational feasibility study. PLoS ONE.

[45] Fridell J.A., Stratta R.J. (2024). Dueling with the dual artery blood supply in pancreas transplantation: Why replace the Y?. Gland. Surg..

[46] Gruessner A.C., Sutherland D.E. (2005). Pancreas transplant outcomes for United States (US) and non-US cases as reported to the United Network for Organ Sharing (UNOS) and the International Pancreas Transplant Registry (IPTR) as of June 2004. Clin. Transplant..

[47] Hill M., Garcia R., Dunn T., Kandaswamy R., Sutherland D.E., Humar A. (2008). What happens to the kidney in an SPK transplant when the pancreas fails due to a technical complication?. Clin. Transplant..

[48] Norman S.P., Kommareddi M., Ojo A.O., Luan F.L. (2011). Early pancreas graft failure is associated with inferior late clinical outcomes after simultaneous kidney-pancreas transplantation. Transplantation.

[49] Weiss A.S., Smits G., Wiseman A.C. (2009). Twelve-month pancreas graft function significantly influences survival following simultaneous pancreas-kidney transplantation. Clin. J. Am. Soc. Nephrol..

[50] Ji M., Wang M., Hu W., Ibrahim M., Lentine K.L., Merzkani M., Murad H., Al-Hosni Y., Parsons R., Wellen J. (2022). Survival After Simultaneous Pancreas-Kidney Transplantation in Type 1 Diabetes: The Critical Role of Early Pancreas Allograft Function. Transpl. Int..

